# Low Dose Furazolidone for Eradication of *Helicobacter pylori* Instead of Clarithromycin: A Clinical Trial

**DOI:** 10.5539/gjhs.v7n1p235

**Published:** 2014-08-31

**Authors:** Aliakbar Hajaghamohammadi, Seyyed Hossein Safiabadi Tali, Rasoul Samimi, Sonia Oveisi, Amir Mohammad Kazemifar

**Affiliations:** 1 School of Medicine, Qazvin University of Medical Sciences, Qazvin, Iran

**Keywords:** *Helicobacter pylori*, furazolidone, non- ulcer dyspepsia, peptic ulcer

## Abstract

**Background:**

*Helicobacter pylori* infection is a common chronic human bacterial infection. Triple- therapy regimen containing a proton pump inhibitor, clarithromycin, and either amoxicillin or metronidazole is commonly used as first-line treatment for its eradication. However, it may not provide the acceptable eradication rate. The present study was designed to evaluated efficacy of low dose furazolidone with amoxicillin and omeprazole for eradication of *H. pylori*.

**Materials and Methods:**

One hundred twenty patients with non- ulcer dyspepsia or peptic ulcer confirmed by upper GI endoscopy, plus *H. pylori* infection confirmed by rapid urease test were included in the study. They were randomly divided into two groups, and then received clarithromycin, amoxicillin, and omeprazole, or furazolidone (100 mg PO bid), amoxicillin, and omeprazole. They were evaluated using urea breath test at the end of the study.

**Findings:**

The eradication rates were 57.6% and 78.8% in clarithromycin and furazolidone groups, respectively. Their difference was statistically significant (P value 0.013). No side effect was seen in the furazolidone group.

**Conclusion:**

Low dose furazolidone rather than clarithromycin can be used as low- cost and effective drug for eradication of *H. pylori*, in combination with amoxicillin and omeprazole.

## 1. Introduction

*Helicobacter pylori* (*H. pylori*) infection is one of the most common chronic human bacterial infections. It is responsible for the most frequent and persistent bacterial infections worldwide. *H. pylori* infection affects nearly half of the world’s population (Garza-González, Perez-Perez, Maldonado-Garza, & Bosques-Padilla, 2014). The consequences of the infection are chronic gastritis, gastric and duodenal ulcers, gastric cancer, and primary gastric lymphoma of mucosa-associated lymphoid tissue type (MALT lymphoma). Eradication of *H. pylori* may cure dyspepsia, peptic ulcer disease, and MALT lymphoma (Peedikayi, AlSohaibani, & Alkhenizan, 2014).

The treatment programs for the implementation of *H. pylori* eradication therapy should be based on patient compliance, antibiotic medication history, and local antibiotic resistance. The common treatment programs included triple therapy, quadruple therapy (containing bismuth), sequential therapy, concomitant therapy with proton pump inhibitor (PPI) and amoxicillin, clarithromycin and metronidazole for 10–14 days (Rongli & Liya, 2014). However, *H. pylori* eradication treatments following these regimens produce cure rates lower than 80%, mainly due to an increase in clarithromycin resistance (Chuah, Tsay, Hsu, & Wu, 2011; Ayala, Escobedo-Hinojosa, de la Cruz-Herrera, & Romero, 2014). Multiple bacterial factors are influencing eradication therapy success rate, with the development of resistance to antibiotics as the most important. Moreover, poor patient compliance due to adverse reactions to the medications, the cost of the drugs, or patient difficulties complying with the therapy regimen should not be ignored (Song &Ang, 2014). Recently, the efficacy of triple therapy decreased globally due to the increased rate of clarithromycin resistance (Talebi Bezmin Abadi, 2014). A recent multicenter study showed that resistance rates have been 17.5% for clarithromycin, 14.1% for levofloxacin and 34.9% for metronidazol (Kanizaj & Kunac, 2014). So, investigation for other treatment regimens has been noticed (Federico, Gravina, Miranda, Loguercio, & Romano, 2014).

Furazolidone is a synthetic nitrofuran derivative with bactericidal or bacteriostatic activity against Gram-positive and Gram-negative bacteria. It is well absorbed in the intestine with no tissue accumulation. It has been used for eradication of *H. pylori* in earlier studies in combination with other drugs as second or third line therapy (Isakov, Domareva, Koudryavtseva, Maev, & Ganskaya, 2002; Eisig et al., 2005; Machado, da Silva, & Viriato, 2008; Cheng & Hu, 2009). One of the great impediments regarding the use of furazolidone is its association with significant adverse effects (Isakov et al., 2002; Eisig et al., 2005; Eisig et al., 2009). Then, can we use lower dose of the drug to avoid its side effects?

The present study was designed to evaluate efficacy of a lower dose of furazolidone in eradication of *H. pylori* as first-line therapy in patients with peptic ulcer disease and non-ulcer dyspepsia compared to clarithromycin.

## 2. Materials & Methods

### 2.1 Materials Studied

One hundred twenty patients older than 15 years were chosen from endoscopy ward of a teaching hospital in Qazvin City, Iran for the study. They were referred for upper GI endoscopy by their treating physicians due to GI symptoms. Rapid urease test had been performed for them and were positive. Their endoscopic diagnosis was peptic ulcer disease or non-ulcer dyspepsia. Any patient with past history of previous GI surgery, cigarette smoking, pregnancy, hemolytic anemia, or need for administration of any antibiotic or non-steroidal anti-inflammatory drugs were excluded from the study.

### 2.2 Methods

The patients were randomly divided into two groups. The first group received clarithromycin 500 mg PO bid and amoxicillin 500 mg PO bid for 2 weeks, in addition to omeprazole 20 mg PO bid for 6-8 weeks. The second group was given furazolidone 100 mg PO bid and amoxicillin 500 mg PO bid for 2 weeks, as well as omeprazole 20 mg PO bid for 6-8 weeks.

The patients were evaluated for eradication of *H. pylori* 2 weeks after ending of the study by urea breath test (UBT).

The study had been approved by local ethical committee of Qazvin’s university of medical sciences. The patients provided written informed consent for participation in the study.

The collected data were analyzed by T-test and chi-square test using SPSS statistical software version 16.0.

## 3. Results

Fifty nine patients were allocated to the first group and sixty one patients were assigned into the second group. All the patients ended their instructed drug regimen and no loss of follow up were seen. Moreover, no disturbing adverse effects of the drugs were reported by the patients. The general characteristics of the groups were demonstrated in Table 1.

**Table 1:** The general characteristics of the studied groups

Age (years)		Group 1	Group 2	P value
	
		41.51±12.11	41.33±13.52	Not significant
**Gender**	male	25	25	Not significant
	female	34	36	
**Level of education**	High school	41	36	Not significant
	Bachelor degree	18	21	
	Higher than bachelor degree	0	4	
**Symptoms**	Epigastric burning pain	6	7	Not significant
	Abdominal pain	32	33	
	Epigastric fullness	21	21	
**Diagnosis**	Non-ulcer dyspepsia (NUD)	48	42	Not significant
	Peptic ulcer (PU)	11	19	

Result of urea breath test 2 weeks after the study was shown in table 2. The results were also evaluated between the groups according to the diagnosis

**Table 2:** Result of urea breath test in the studied groups 2 weeks after the study

	Group 1			Group 2			P value

	NUD	PU	total	NUD	PU	total	
UBT negative	27(56.3%)	7(63.6%)	34(57.6%)	33(78.6%)	15(78.9%)	48(78.7%)	0.013 Odds ratio 2.715 (95% CI: 1.218-6.050)
UBT positive	21(43.7%)	4(36.4%)	25(42.4%)	9(21.4%)	4(21.1%)	13(21.3%)	

## 4. Discussion

The present study suggested that furazolidone can be use as first line drug for eradication of *H. pylori* instead of clarithromycin. We reached the eradication rate near 80%, even though we have used low dose furazolidone (100 mg per dose rather than 200 mg per dose that had been used in earlier similar studies).

There are many schemes for treating *H. pylori* infection. However, an optimal treatment has not been defined, and there is not a single antibiotic treatment that can eradicate it (Garza-González et al., 2014). Undeniably, the *H. pylori* treatment story is far from complete. The current sharp fall in eradication rate of therapy has sobered to be careful in this topic (Talebi Bezmin Abadi, 2014). The effectiveness of the most commonly used therapies has been increasingly compromised by the rapid emergence of antibiotic-resistant strains of *H. pylori* and by poor adherence to treatment by patients (Garza-González et al., 2014). The reason for the decrease in the efficacy of standard triple therapy is mainly due to the increase in the resistance against clarithromycin (lee & Kim, 2014). In general, several international guidelines for treating patients diagnosed with *H. pylori* infections are consistent with the use of triple therapy as the first-line treatment. This treatment consists of the administration of a proton pump inhibitor (PPI), clarithromycin, and amoxicillin for 7-14 d (Ayala G et al., 2014). However, recent reports indicate that this regimen does not achieve acceptable eradication rate. The most important cause for the reduced success of standard triple therapy is the increasing rate of *H. pylori* clarithromycin/ metronidazole resistance (Peedikayi et al., 2014). The contributory factors to treatment failure are multidimensional and complex. Host genetic factors, *H. pylori* virulent factors, antibiotic resistant *H. pylori* strains, smoking habits, compliance to therapy and duration of therapy all affect the treatment outcome (Song & Ang, 2014). Clinical experience in Iran has demonstrated that the eradication rate of *H. pylori* with the standard therapy is much lower than the rate reported from western and developed countries, and a high rate of metronidazole resistance (37%) and an increasing rate of resistance to clarithromycin have emerged (Ghadir et al., 2011). So, research for more suitable therapy necessitates.

Eisig and his colleagues (2005) have used quadruple therapy consisted of furazolidone, tetracycline, omeprazole, and bismuth for 7 days in patients with previous treatment failure. They have reported 67% eradication rate. He has studied another drug combination with furazolidone, levofloxacin, and lansoprazole in his later research, again in patients with previous treatment failure (Eisig et al., 2009). He has reported 88% eradication rate with this regimen. Isakov and his coworkers (2002) have evaluated efficacy of furazolidone, tetracycline, and omeprazole in eradication of *H. pylori* in patients with first-line treatment failure. They reached to 90% eradication rate. Combination of furazolidone and amoxicillin plus omeprazole has been evaluated by Felga and his collaborators (2008) in patients with first-line treatment failure. They have reported 68% eradication rate. The above mentioned studies have been performed on patients with previous treatment failure. All of them had used furazolidone 200 mg twice a day for 1 to 2 weeks. However, the main concern regarding use of furazolidone is the adverse effects observed, especially among individuals who do not adhere to the dietary restrictions recommended, since this drug belongs to the group of the MAO inhibitors. So, we used half of this dose in our study. We wanted to clarify the eradication rate with this dosage of the drug. This dosage has been used by Cheng and Hu (2009) in combination with amoxicillin, bismuth, and rebaprazole with the reported 82% eradication rate. The present study reached to the nearly same rate, though we did not used bismuth, and used omeprazole instead of rebaprazole. The eradication rate in the present study is comparable to studies of Eisig (2009), Isakov (2002), and Felga (2008); despite the fact that we had used half-dose furazolidone in combination with the low cost drugs (amoxicillin and omeprazole). The eradication rate was higher than that of the higher cost drug, clarithromycin.

## 5. Conclusion

The present study suggests that low- dose furazolidone can effectively be used for treatment of *H. pylori* to prevent the side effects of the drug.
